# To Prevent Alloying of Crowns by Fusible Metal

**Published:** 1906-07

**Authors:** 


### TO PREVENT ALLOYING OF CROWNS BY FUSIBLE METAL.

First, never use it. It so happens, however, that many prefer this method of shaping shells, and to get rid of the metal throw shell and its low-melting contents into a basin of boiling water, the basin still over the flame; or worse, they heat the shell in the flame itself. In the first instance the shell is in contact with the bottom of the basin, and for the time being becomes a part of that utensil; as a consequence it drinks in a small quantity of the fusible-metal die, and notwithstanding its treatment later to a bath in acid it is alloyed to some degree and for all time. In the second instance, no matter how skillful the workman, some "dross" clings to the inner surface of the crown; its perfect removal is next to impossible. The effect of this maltreatment shows in the melting down of the alloyed part during annealing or reinforcing with solder; or, if it escape this fatality, in the "greening" of the crown when subjected to the action of the secretions, especially of some mouths. The greenish gold crowns, originally twenty-two karat, have all been colored in this way. If one must use fusible metal forms, and especially of a melting point but little below the temperature of boiling water, contact with the basin may be prevented by covering the bottom with a thickness or two of blotting paper, or with a layer or two of cloth. In every case the shell should be thrown into acid and kept there until all traces of the baser metals have been removed.—*Office & Laboratory.*

